# Retrospective Cohort Study to Assess the Risk of Rabies in Biting Dogs, 2013–2015, Republic of Haiti

**DOI:** 10.3390/tropicalmed2020014

**Published:** 2017-06-12

**Authors:** Alexandra M. Medley, Max Francois Millien, Jesse D. Blanton, Xiaoyue Ma, Pierre Augustin, Kelly Crowdis, Ryan M. Wallace

**Affiliations:** 1 Centers for Disease Control and Prevention, Atlanta, GA 30329, USA; asi5@cdc.gov (J.D.B.); hjv4@cdc.gov (X.M.); euk5@cdc.gov (R.M.W.); 2 Ministry of Agriculture and Natural Resources and Rural Development, Port-au-Prince, Haiti; maxfrancoismillien@gmail.com (M.F.M.); pdilius@yahoo.fr (P.A.); 3 Christian Veterinary Mission, Port-au-Prince, Haiti; crowdisk@yahoo.com

**Keywords:** rabies, post-exposure-prophylaxis (PEP), surveillance, risk assessment, dog bite, Haiti, retrospective cohort

## Abstract

Background: In canine rabies endemic countries the World Health Organization recommends post-exposure prophylaxis (PEP) be initiated immediately after exposure to an animal suspected to have rabies. Limited capacity in low and middle income countries to assess biting animals for rabies may result in the over prescription of rabies biologics. Few guidelines exist to determine the risk of whether a dog that has bitten someone is rabid. Given PEP cost and access limitations in many countries, accurate and timely assessment of dogs that have bitten people may reduce unwarranted PEP use and improve healthcare seeking behaviors. Methods: Haiti’s animal rabies surveillance program utilizes veterinary professionals to conduct rabies assessments on reported biting dogs and records characteristics of the dog, health outcomes, and laboratory results in a national database. Characteristics of rabid dogs were assessed through a retrospective cohort study of biting dogs investigated during the period from January 2013–December 2015. 1409 biting dogs were analyzed; 1361 dogs that were determined to not have rabies were compared to 48 laboratory-confirmed rabid dogs. Rate ratios, sensitivity, specificity, positive predictive values, negative predictive values, likelihood ratios, quarantine survival of biting dogs, and a risk matrix were developed. Findings: The assessor’s determination that the animal likely had rabies was the most significant predictive factor for a rabid dog (RR = 413.4, 95% CI 57.33–2985, Sn = 79.17, Sp = 91.92). Clinical factors significantly associated with rabid dogs included hypersalivation, paralysis, and lethargy (RR = 31.2, 19.7, 15.4, respectively). Rabid dogs were 23.2 times more likely to be found dead at the time of the investigation compared to case negative dogs (95% CI 14.0–38.6). Rabid dogs were also significantly more likely to lack a history of rabies vaccination or be unowned (RR = 10.3 95% CI 2.5–42.3 and RR = 4.5 95% CI 2.0–10.1, respectively). Rabid dogs were four times more likely to have bitten multiple people (RR = 4.0 95% CI 1.9–8.3). Most rabid dogs died or were killed before quarantine (75%) and all died by day 3 of quarantine, compared to <1% of quarantined case-negatives. The greatest risk of death was predicted to be for persons bitten on the head or neck from symptomatic dogs. Bites from dogs deemed healthy by veterinary assessors and which were available for quarantine presented less than a 0.05% risk of rabies death to the victim. Conclusions: Vaccination of all persons exposed to a suspected rabid dog is a highly effective approach to minimize human rabies deaths. However, this may place undue financial burden on bite victims that have had a low-risk exposure and over-prescription may contribute to regional supply shortages. The results here indicate that in a low-resource country such as Haiti, a well-trained veterinary assessor can provide an accurate risk assessment of biting dogs based on a standard case investigation protocol. In canine rabies endemic countries with limited access to PEP, or where PEP costs may cause undue burden on bite victims, structured risk assessments by trained professionals may be a reliable method of triaging PEP for bite victims. Evaluating rabies risk through a matrix of bite location and risk factor in the dog presents a clear delineation of high and low risk encounters and should be used to develop data-derived PEP recommendations.

## 1. Introduction

Rabies is a zoonotic disease responsible for at least 2.5 million human rabies deaths over the past century [1]. The overwhelming majority of human cases are the result of a bite from a rabid dog [1,2]. Rabies is vaccine-preventable; however, in the absence of appropriate post-exposure prophylaxis (PEP) the rabies virus induces an acute neurologic illness followed by inevitable death. Canine rabies is endemic in the Republic of Haiti, where an estimated 130 human rabies deaths occur annually [1,3]. Persistent human rabies deaths are due to a culmination of factors often seen in low and middle income countries: low dog vaccination coverage, low awareness, and lack of access to PEP in rural areas, all of which are barriers encountered in Haiti [3]. 

Until the rabies virus is eliminated in the reservoir population, humans will remain at risk of exposure. In rabies endemic countries, appropriate and timely wound management and PEP are critical for preventing human rabies deaths [4]. The World Health Organization (WHO) provides recommendations for rabies post-exposure prophylaxis after a dog bite in a canine rabies-endemic country but the recommendations leave ambiguity in interpretation [5]. These recommendations state that a risk assessment should be performed when considering initiating PEP [6]. However, they also recommend that “prophylaxis should be instituted immediately” and “continued while awaiting laboratory results or during the observation period” [6]. Information regarding how to assess rabies risk in a biting animal, and when assessment results should impact initiation of PEP, are not explicit in this WHO document. 

While Haiti is considered endemic for rabies, results from a multi-year surveillance program revealed that only 1%–5% of biting dogs had rabies [3]. Considering current WHO recommendations, this combination of a low rabies prevalence in biting dogs and a risk-averse, conservative recommendation for PEP initiation may result in over-prescription of rabies biologics [7]. In low and middle income countries, the cost of PEP in relation to income can be quite burdensome to households [8]. As many as five rabies vaccine doses over 4 weeks are required, placing a burden on those who cannot afford the travel or time off of work, let alone the cost of biologics [6]. Furthermore, there is an unmet need in many countries for safe and accessible PEP [5]. This warrants investigation into how limited and expensive rabies biologics can be used as efficiently as possible while ensuring that truly exposed bite victims seek medical care for prevention of this 100% fatal disease.

The need to characterize low-risk rabies exposures has become an increasingly important issue in Haiti, where a government-operated post-bite dog investigation program resulted in an 85% increase in the detection of persons with dog bites [3]. As Haiti has adopted the WHO rabies exposure recommendations, this increase in bite detection significantly increased PEP utilization and costs for bite victims [9,10]. Undurraga et al. reported in 2017 that delaying PEP when the risk of rabies was low, and when the dog was available for quarantine, could reduce PEP costs without imposing undue risk to bite victims in Haiti [9]. 

This community based animal rabies surveillance program, Haiti Animal Rabies Surveillance Program (HARSP), employs veterinary professionals to investigate biting dogs, conduct a rabies assessment, quarantine or euthanize, and provide rabies counseling to bite victims [3]. Under this type of structured program, the possibility for standardized risk assessment has become possible, but there is an unmet need to specify what WHO considers to be a low-risk exposure. This study provides a descriptive analysis of the characteristics of rabid versus non-rabid dogs that have bitten humans. The purpose of this study was to evaluate whether a risk assessment conducted by veterinary professionals in a program such as HARSP could reliably approximate the risk of rabies in a biting dog, and to generate evidence for the classification of low-risk exposures in the canine rabies endemic setting.

## 2. Methods 

### 2.1. Data Set and Cohort Selection

Data analyzed for this study were made available from the Haiti Ministry of Agriculture from the national HARSP database (2013–2015). HARSP assessors are trained veterinary agents from the Ministry of Agriculture, Natural Resources, and Rural Development (MARNDR) and undergo a one-week field training program conducted by the CDC and the Christian Veterinary Mission, and must pass the Global Alliance for Rabies Control’s Rabies Educator Certification program [11,12]. HARSP has standard investigation protocols and investigation forms (see Supplementary Materials) for which veterinary agents must display proficiency. New assessors conduct job shadowing for 2–4 weeks. Each department has one lead technician who typically has two years of agriculture-focused education at the University level and is responsible for compiling reports for central collection. 

Assessors receive reports of animals that are suspected to have rabies from the Ministry of Health, hospitals, veterinarians, and directly from community members. Assessors respond within 24 h to assess the animal, make a determination as to whether to pursue a 14-day quarantine or euthanasia, and to counsel the bite victim on appropriate post-bite wound care. Animals considered symptomatic for rabies are immediately euthanized and submitted to the national laboratory for testing. All samples are confirmed by the direct fluorescent antibody test. Results are validated bi-annually at the United States Centers for Disease Control and Prevention (CDC) to ensure diagnostic proficiency. Standardized data are collected and entered into a national database (Microsoft Access). At the conclusion of an investigation, each dog is classified as 1-confirmed case, 2-probable case (clinical case definition), 3-suspect case (unavailable for assessment), 4-case negative (14-day quarantine or negative direct fluorescent antibody result) [3]. All investigation forms and case determinations are reviewed by a program manager weekly.

Data for the time period from 1 January 2013 to 31 December 2015 were used for analysis. The analysis was limited to dogs involved in a human bite event for which diagnostic test results or quarantine results were recorded (classifications 1 and 4). Dogs classified as probable or suspect rabies cases were excluded. The final cohort analyzed comprised 48 rabies positive dogs and 1361 dogs for which rabies was ruled out (total *n* = 1409).

### 2.2. Evaluation of Single Variables

HARSP surveillance data contained twelve stratified and binary variables that were considered in this study to represent various demographics and clinical signs present in rabid dogs: entity reporting the bite, ownership status of the dog, the number of individuals bitten, dog’s sex, dog’s age, presence of aggression, hypersalivation, paralysis, lethargy, vaccination status of the dog, condition of the dog upon location, and the decision of the trained veterinary agent (also referred to as the assessor). Further details on each variable are described in Table 1. Risk ratio (RR) and corresponding 95% confidence interval (CI), sensitivity (Sn), specificity (Sp), positive predictive value (PPV), negative predictive value (NPV), positive likelihood ratio (PLR), and negative likelihood ratio (NLR^−1^) of each variable were calculated, using both Microsoft Excel and CDC Epi Info software. Risk ratios were calculated for variables with greater than 2 strata against an assigned reference group within that variable.

### 2.3. Evaluation of Mortality during Quarantine

All case negatives (*n* = 1361) were compared to confirmed rabies cases (*n* = 48) to determine how many died before and during quarantine. The time period between the date of report and the date of death—regardless of quarantine status—was used to calculate the variable corresponding to the number of days until a dog died. The date of death is the date that the dog was reported to have died, was found dead by a veterinary agent, was killed (by the public or in quarantine for humane purposes), or died by natural causes (rabies or other disease). The percent of biting dogs which were alive was calculated on a daily basis for a total of 14 days, the quarantine duration used for biting dogs in the Republic of Haiti.

### 2.4. Risk Matrix

A risk matrix was created to assess the probability of dying from rabies based on the physical location of the dog bite and the characteristics of the biting dog. The probability of rabies in dogs was calculated for selected high and low-risk variables assessed in this study. The probability of rabies death was obtained by the product of the two variables: probability of rabies in the biting dog and probability of death based on location of exposure. Probability of death by exposure was obtained from Babes et al. [13].

## 3. Results

**Figure 1:**
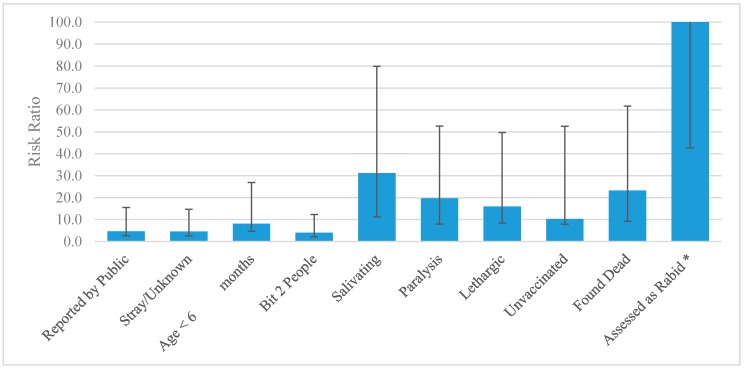
Risk ratio of significant variables and their 95% CI. * “Assessed as rabid” has a RR = 413 and 95% CI upper limit = 2985. For ease of comparison to other variables in this figure, it is limited to RR = 100 and no upper limit is shown.

**Figure 2:**
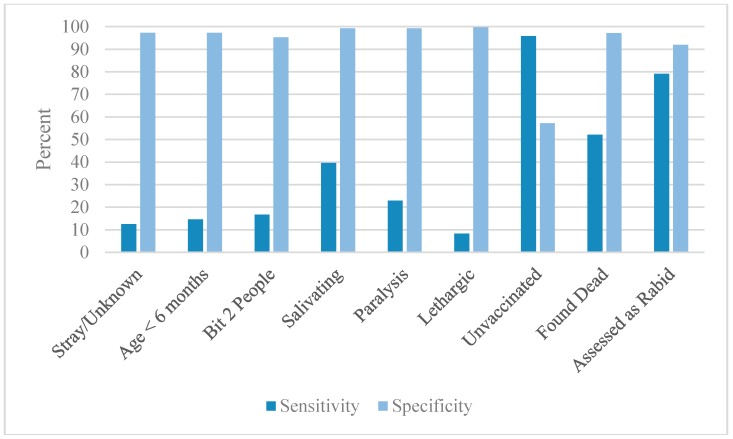
Sensitivity and specificity of the relevant variables.

1409 animals were eligible for this retrospective cohort analysis. Rabies was confirmed in 48 animals and ruled out in 1361. Suspect and probable cases were excluded from the study, as no definitive case determination could be assigned (probable cases, *n* = 42 and suspect cases, *n* = 265). Of the rabies-positive dogs included in the study, 29 (60.4%) were dead at the time of investigation, 7 (14.6%) were euthanized on the day of the investigation, and 12 (25%) were placed into quarantine. Table 2 and Figure 1 show risk ratios and 95% confidence intervals. Table 3 and Figure 2 show the Sn, Sp, PPV, NPV, PLR and NLR^−1^ of all variables.

### 3.1. Single Variable Results

The assessor’s determination that the animal likely had rabies was the most significant predictive factor for a rabid dog (RR = 413.4, 95% CI 57.33–2985). Clinical factors significantly associated with rabid dogs included hypersalivation, paralysis, and lethargy (RR = 31.2 95% CI 20.0–48.7, 19.7 95% CI 11.7–33.0, and 15.9 95% CI 7.5–33.8, respectively). Dogs that were dead at the time the assessor arrived for investigation were 23.2 times more likely to have rabies compared to those that were alive (95% CI 14.0–38.6). Rabid dogs were 8.1 times more likely to be puppies than adult dogs (95% CI 3.5–18.8). Dogs lacking a history of rabies vaccination and unowned dogs were significantly more likely to be rabies positive (RR = 10.3 95% CI 2.5–42.3 and RR = 4.5 95% CI 2.0–10.1, respectively). Rabid dogs were four times more likely to have bitten multiple people (RR = 4.0 95% CI 1.9–8.3). Sex was the only variable assessed in this study that did not display an association with rabid dogs. Aggressive behavior was protective (RR < 1). However, this dataset is limited to biting dogs, therefore this should be interpreted cautiously. 

When analyzed independently, the sensitivity of most variables was low. Of those that were above 50%, a lack of rabies vaccination was the highest (Sn = 97.8%). Other notable variables with good sensitivity were the rabies determination of the assessor and when the dog was found dead at the time of investigation (Sn = 79.2% and 52.1%, respectively). In contrast, most variables had a specificity >90%. The notably low specificities were the vaccination status (57.2%) and the presence of aggression (6.4%). 

Likelihood ratios indicate the probability that a biting dog with the presence of a particular variable is rabies positive, compared to a biting dog that does not have the presence of that variable [14]. Likelihood ratios were interpreted as follows: >4 indicated a 25% increase in probability of an outcome (moderate), and those >10 indicated a 45% increase in probability of an outcome (large) [15,16]. The NLR^−1^ results show a moderate increase in the probability that a rabid dog will be classified as rabid by a veterinary agent compared to case negatives (4.8), and a large increase in the likelihood that a biting dog is rabid if it has an unknown or no vaccination history (13.7). The PLR results indicate a moderate increase in the probability of rabies for stray/unknown dogs (4.6) and puppies (5.4), and a large increase for hypersalivation (53.8), paralysis (31.2), lethargy (28.4), and being found dead (18.7). A large increase in probability was found in the PLR for both assessor decisions of a dog that has rabies (102.4) and does not have rabies (14.5).

### 3.2. Mortality and Quarantine Results

**Figure 3:**
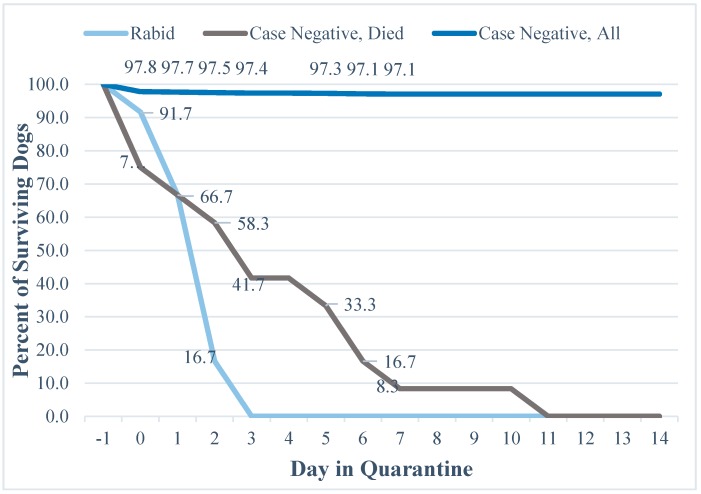
Proportion of surviving dogs during a 14-day rabies quarantine.

Twenty-four of the total cohort died during the quarantine period, of which 12 tested positive and 12 tested negative for the rabies virus. The majority of rabid dogs were dead at the time of investigation (*n* = 36, 75%), and of the 12 dogs quarantined, 100% were dead by the third day of quarantine. The average duration until death for a rabid dog that was placed in quarantine was 1.75 days (Figure 3). Forty-two of the case-negatives died before a quarantine was issued (3.1%). Assessors placed 1319 of the case-negatives into quarantine, of which 1307 (99.1%) were healthy after 14 days, and 12 died (0.9%). The average duration until death for a case-negative dog that was placed into quarantine was 3.67 days, 2.1 times longer than for rabid dogs (See Supplementary Materials for the table of values that correspond to Figure 3).

## 4. Discussion

### 4.1. Objective of the Study

Although untreated rabies infections are 100% fatal, vaccination of all persons bitten by a dog in a canine rabies endemic country is a highly conservative approach to minimize rabies cases, and it may place an undue burden on low-risk exposure bite victims while also contributing to regional vaccine supply shortages [7,9,10]. Few studies describe the risk of rabies in biting dogs in low and middle income countries, and global recommendations provide little guidance on how biting animals should be assessed. A 2005 study by Tepsumethanon et al. in Thailand looked at six criteria in living dogs to determine rabies status, with all but one of the variables focusing on clinical signs and disease course [17]. This algorithm was reported to have high Sn and Sp (Sn = 90.6%, Sp = 96.0%), establishing the precedent that risk assessments may be a reliable method for determining PEP recommendations. However, the Tepsumethanon evaluation must be completed over a 10-day period, making the results incompatible for determining risk for the purpose of PEP initiation [5,17]. This study included rabies positive and case negative dogs, but excluded dogs that were defined as suspect or probable. This selection bias was essential, because determining defining characteristics of rabid versus non-rabid dogs requires a definitive diagnosis. In addition, there is likely a contingent of dogs who are not reported, and therefore the data set is limited to only those biting dogs that have been assessed by veterinary agents. It is possible that this data set does not fully represent the biting dog population, although it is not feasible to say for certain how excluding these dogs would affect the results. The data presented here account the characteristics of over 1400 biting dogs and show that some factors collected as part of a post-bite rabies risk assessment are highly predictive for rabies and could be used to inform decisions to initiate a rabies PEP series.

### 4.2. Characteristics of Rabid Dogs

While it is commonly assumed that biting dogs in canine-rabies endemic countries present a high risk for rabies, this study of a post-bite surveillance system found that only 3.4% of biting dogs are actually affected with rabies. Haiti is largely considered to have one of the highest rates of canine rabies, globally. Therefore, this should not be interpreted as evidence of a low rate of enzootic rabies transmission, rather this is likely a reflection of the frequent and continuous bite exposures that occur in countries that value dog ownership, which obscures the lower rate of continuous bite exposures from rabid dogs. This low frequency of rabies among biting dogs in Haiti supports the need to develop risk assessment criteria that can reliably identify low-risk situations in which it would present little to no risk to delay PEP during a quarantine period.

This is the largest evaluation of characteristics of biting dogs in a canine-rabies endemic country, and establishes common characteristics that increase the likelihood that a biting dog is affected with the rabies virus. To little surprise, dogs with symptoms consistent with rabies were at higher risk of being confirmed rabid (hypersalivation, paralysis and lethargy, RR = 31.2, 19.7, 15.9 respectively). Even more significant predictors that a biting dog would have rabies were a lack of previous rabies vaccination and when the dog was dead at the time of the post-bite investigation. However, the greatest risk factor for predicting whether a biting dog was or was not rabid, was not a clinical sign, but rather the subjective opinion of the assessor. An assessor declaring an animal as ‘rabid’ increased the likelihood of rabies 400-fold. This variable has no concrete, reproducible definition, rather it is the assessor’s overall judgment after considering a combination of clinical factors, vaccination history, and the circumstances surrounding the bite event. Other variables such as age, number of people bitten, and ownership status were significantly associated with rabies among biting animals, but to a lesser degree. The findings presented here confirm that it is possible to identify rabid animals, with high confidence, based on a basic health evaluation by trained veterinary professionals.

### 4.3. Decision Making Based on the Variables

Sensitivity refers to a test’s ability to identify positive cases, and a high sensitivity would result in few rabid dogs being misclassified as case-negative [18]. Rabies is an invariably fatal disease if PEP is not initiated in a timely manner, therefore it is more prudent to consider the sensitivity of variables in a risk assessment for PEP determination. Relying on variables with low sensitivity for PEP recommendations could result in a proportion of bite victims being incorrectly told that rabies was not a risk, PEP would incorrectly be delayed or not given, and these bite victims would risk death. Relying on variables with low specificity for PEP recommendations could result in a proportion of bite victims being incorrectly told that they had a rabies exposure, PEP would be given unnecessarily, and these bite victims would have undergone an unnecessary expense. Clearly, when considering the lethality of rabies, variables with high sensitivity should be prioritized during a risk assessment. 

Considering that rapid and reliable rabies diagnostic capacity is lacking in most rabies endemic countries, risk assessments and quarantine periods may be the only tools available to guide PEP recommendations [19]. For this analysis we considered the variables collected in HARSP’s risk assessment as ‘diagnostic evaluations’ and compared them to a rabies outcome (i.e., rabid vs. case-negative). In this respect, the diagnostic sensitivity of most risk assessment variables was fairly low (on average, between 30% and 50%). Many variables, when considered alone, would not be reliable for determining rabies risk and influencing PEP decisions. Only vaccination status and assessor decision had relatively high sensitivities (95.8% and 79.2%, respectively) when compared to the Tepsumethanon criteria (91%) [17]. The ideal variable for determining rabies PEP need would be 100% sensitive, which no single variable achieved. However, the objective of this study was to determine situations that present low-risk, not no-risk, and to identify scenarios in which PEP could be safely delayed. Given that all rabid animals died within 3 days of quarantine, reduced sensitivity of risk assessment variables may be tolerable when the dogs are available for quarantine. Certain variables stand out as having multiple significant diagnostic properties. Unowned dogs and puppies were moderately diagnostic, with a fairly high RR, Sp, and PPV. Stronger still were the three clinical signs of lethargy, paralysis, and hypersalivation, which had high RR, Sp, PPV, and PLR. Dogs that were dead at the time of the bite investigation, and those without a documented rabies vaccination history, also displayed good diagnostic performance. However, the most important diagnostic test considered in this analysis was the rabies determination made by the assessor, which displayed consistent prognostic value across the single variables. (Sn = 79.2%, Sp = 91.9). 

Consideration of the interactions of multiple variables may help further clarify a classification scheme to gauge low-risk exposures, as has been done for other diseases that require treatment before laboratory diagnosis is possible [15,20,21]. In the case of rabies, this would mean identifying a combination of easily assessable variables at the time of the bite incident for the purpose of immediate PEP decisions. There are also benefits to quick assessment of high risk exposures, as this leads to prompt healthcare seeking behaviors as well as increases adherence to treatment recommendations. 

In addition to aiding risk assessment decisions, some variables may provide critical data for monitoring rabies programs. For example, in Haiti a large proportion of rabid dogs were unowned, which may influence policy makers to promote responsible dog ownership. While some variables have a clear impact on risk assessments, others may act as programmatic and policy indicators. 

### 4.4. Evaluation of Mortality and Quarantine Data

During this study period, HARSP veterinary agents only misclassified one rabid dog as probably not having rabies at the time of the risk assessment (2.1%). In this instance the dog was quarantined and died 3 days later, suggesting that the quarantine could serve as a secondary measure to gauge rabies risk [4,22]. It is not surprising that all the confirmed rabid animals died in quarantine, but it is interesting and of relevance that 75% died before the quarantine was instituted, and that the remaining dogs died within three days of placement in quarantine. Death took over a week for the majority of case-negative dogs that died in quarantine. Of all the case negative dogs that entered quarantine, over 99% were still alive by day 14. This study evaluated factors that could lead to an immediate risk assessment determination to influence PEP decisions. This data would indicate that even if a risk assessment were misclassified after a bite, rabid dogs are likely to die early during the quarantine period and this early death event should trigger a re-assessment of rabies risk. 

It has been well established that a dog that is shedding the virus will show signs of illness within a 10-day time period [2,22,23,24,25]. This finding was supported by the results of this study; all rabid dogs died within 3 days of quarantine. Given both the historical findings, and findings from this study, a 10-day post-bite quarantine is supported in dogs. Programs, such as in Haiti, utilizing longer quarantine periods may be inefficiently utilizing resources that could be diverted to post-bite follow up of dogs or dog vaccination.

### 4.5. Risk Matrix

The risk of developing rabies from a dog bite is dependent on multiple factors including the epidemiology of rabies in the country, the type of exposure, and the probability the biting animal was infectious. By systematically collecting surveillance data through Haiti’s Integrated Bite Case Management program, the risk of developing rabies can be calculated and presented as a matrix to visualize situations of high and low probability of rabies death (see Table 4). Regardless of the assessment of the biting dog, nearly all head/neck bites were associated with a high probability of death (28–0.04%). Likewise, regardless of the location of exposure, nearly all bites from dogs with symptoms of rabies were associated with the high probability of death (28–0.6%). Contrasting these high-risk settings, the probability of death was very low for most situations of non-penetrating bite exposures and all situations in which the animal was assessed as ‘healthy and available for quarantine’. Tolerance for ‘risk’ is subjective, therefore interpretation of the risk matrix may vary. However, this presents an objective method for beginning to develop algorithms for PEP determination. The risk matrix presented here utilizes data specific to biting dogs in Haiti and may not be representative of all cultural and epidemiologic situations.

## 5. Conclusions

HARSP is a unique integrated bite case management program for low-resource settings that employs para-veterinary professionals dedicated to assessing biting animals. The risk factors identified in this study will act to inform veterinary agents in Haiti as they conduct in the field assessments of biting dogs. These assessors operate under a defined protocol and training regimen. It is unclear whether similar results could be reproduced in other programs where assessors might receive different training or follow different protocols. Therefore, the generalizability of these findings to other country’s programs should be undertaken with attention to these differences. In many developed nations, existing infrastructure allows public health systems to delay PEP in low-risk scenarios while diagnostic testing is pursued [4]. However, in low-resource settings, reliable and timely diagnostic capacity is often not available [3,8,26]. As a consequence, the WHO’s conservative vaccination policy, although prudent given rabies infection has nearly 100% mortality, may result in the over-prescription of PEP. The results here indicate that in a low-resource country, such as Haiti, a well-trained assessor can provide a highly accurate estimate of the rabies risk from a biting dog for the purposes of recommending no treatment or advising high risk victims of the need for timely treatment. Policy makers who wish to divert resources from reactive, PEP-based, prevention to pro-active, dog-vaccination oriented prevention may wish to consider conducting similar risk analyses.

## Figures and Tables

**Table 1:** Summary of variables.

Variable	Strata	Specifics
Entity Reporting the Dog Bite Incident	All Health Sectors	Ministry of Health, Local Health Department, Hospitals
	Veterinary Sectors	Veterinarians and Veterinary Agents
	Public	Notifications originating directly from a community member
Ownership Status	Owned	
	Stray/owner not identified	
Number of People Bitten	1 person bitten	
	2 people bitten	
	3 or more people bitten	
Age	Puppy	<6 months
	Junior	6 months–1 year
	Adult	>1 year
	Unknown	No age reported
Sex	Female	
	Male	
	Unknown	
Aggression	Present or Absent	Aggression is determined by the rabies assessor
Hypersalivation	Present or Absent	Hypersalivation is determined by the rabies assessor
Paralysis	Present or Absent	Paralysis is determined by the rabies assessor
Lethargy	Present or Absent	Lethargy is determined by the rabies assessor
Vaccination Status	Vaccinated	Owner-reported that dog was vaccinated at least once
	Not Vaccinated	Includes unvaccinated and unknown vaccination status
Status of Dog at the Time of Investigation	Alive	
	Dead	Hit by car, killed, died of natural causes
Rabies Assessor’s Decision	Probably not Rabies	
	Probably Rabies	
	Dead/Not Assessed	

**Table 2:** Risk ratios of the 12 dog bite-related variables.

Variable	Test Group	Rabies Positive *n* = 48		Case Negative *n* = 1361		Risk Ratio and 95% CI
		*n*	%	*n*	%	
Entity Reporting the Dog Bite Incident	All Health Sectors ^1^	19	39.6%	1033	75.9%	*REF*
	Veterinary Sectors ^2^	22	45.8%	252	18.5%	4.45 (2.44–8.09)
	Public	7	14.6%	76	5.6%	4.67 (2.02–10.79)
Ownership Status	Owned	42	87.50%	1324	97.28%	*REF*
	Stray or Unknown	6	12.50%	37	2.72%	4.54 (2.04–10.10)
Number of People Bitten	Bit 1	36	75.00%	1260	92.58%	*REF*
	Bit 2	8	16.67%	64	4.70%	4.00 (1.93–8.29)
	Bit ≥ 3	4	8.33%	37	2.72%	3.51 (1.31–9.41)
Sex	Female	14	29.17%	456	33.50%	*REF*
	Male	17	35.42%	759	55.77%	0.74 (0.37–1.48)
	Unknown	17	35.42%	146	10.73%	3.50 (1.77–6.94)
Age	Adult	16	33.33%	803	59.00%	*REF*
	Puppy	7	14.58%	37	2.72%	8.14 (3.53–18.76)
	Junior	17	35.42%	475	34.90%	1.77 (0.90–3.47)
	Unknown	8	16.67%	46	3.38%	7.58 (3.40–22.38)
Aggression	Non-aggressive	9	18.75%	87	6.39%	REF
	Aggressive	39	81.25%	1274	93.61%	0.32 (0.16–0.63)
Salivation	Normal Salivation	29	60.42%	1351	99.27%	*REF*
	Hypersalivation	19	39.58%	10	0.73%	31.18 (19.95–48.73)
Paralysis	Non-paralytic	37	77.08%	1351	99.65%	*REF*
	Paralytic	11	22.92%	10	0.35%	19.65 (11.72–32.95)
Lethargy	Non-lethargic	44	91.67%	1357	99.71%	*REF*
	Lethargic	4	8.33%	4	0.29%	15.92 (7.51–33.75)
Vaccination Status	History of Rabies Vaccination	2	4.17%	434	31.89%	*REF*
	Not vaccinated or unknown history	46	95.83%	927	68.11%	10.31 (2.51–42.26)
Status of Dog at Time of Investigation	Found Alive	23	47.92%	1323	97.21%	*REF*
	Found Dead	25	52.08%	38	2.79%	23.22 (13.99–38.55)
Assessor's Decision	Dog not showing signs of Rabies	1	2.08%	1251	91.92%	*REF*
	Dog likely to be rabid	38	79.17%	77	5.66%	413.4 (57.33–2985)
	Dead/Not Assessed	9	18.75%	33	2.42%	268.1 (34.79–2069)

^1^ Ministry of Health, Local Health Department, Hospitals. ^2^ Veterinarians and Veterinary Agents.

**Table 3:** Sensitivity, specificity, positive predictive value, negative predictive value, negative likelihood ratio, and positive likelihood ratio of the 12 dog bite-related variables.

Variable	Test Group	SENS	SPEC	PPV	NPV	NLR^−1^	PLR
Entity Reporting the Dog Bite Incident	All Health Sectors	39.58%	24.10%	1.81%	91.88%	0.399	0.522
	Veterinary Sectors	45.83%	81.48%	8.03%	97.71%	1.504	2.475
	Public	14.58%	94.42%	8.43%	96.91%	1.105	2.612
Ownership Status	Owned	87.50%	2.72%	3.07%	86.05%	0.217	0.899
	Stray or Unknown	12.50%	97.28%	13.95%	96.93%	1.112	4.598
Number of People Bitten	Bit 1	75.00%	7.42%	2.86%	89.38%	0.297	0.810
	Bit 2	16.67%	95.30%	12.50%	97.01%	1.144	3.544
	Bit ≥ 3	8.33%	97.28%	10.81%	96.78%	1.061	3.065
Sex	Female	29.17%	66.50%	2.98%	96.38%	0.939	0.871
	Male	35.42%	44.23%	2.19%	95.10%	0.685	0.635
	Unknown	35.42%	89.27%	10.43%	97.51%	1.382	3.302
Age	Adult	33.33%	41.00%	1.95%	94.58%	0.615	0.565
	Puppy	14.58%	97.28%	15.91%	97.00%	1.139	5.364
	Junior	35.42%	65.10%	3.46%	96.62%	1.008	1.015
	Unknown	16.67%	96.62%	14.81%	97.05%	1.159	4.931
Aggression	Non-aggressive	18.75%	93.61%	9.38%	97.03%	1.152	2.933
	Aggressive	81.25%	6.39%	97.03%	90.63%	0.341	0.868
Salivation	Normal Salivation	60.42%	0.73%	2.10%	34.48%	0.019	0.609
	Hypersalivation	39.58%	99.27%	65.52%	97.90%	1.643	53.873
Paralysis	Non-paralytic	77.08%	0.73%	2.67%	47.62%	0.008	0.027
	Paralytic	22.92%	99.27%	52.38%	97.33%	1.288	31.190
Lethargy	Non-lethargic	91.67%	0.29%	3.14%	50.00%	0.035	0.919
	Lethargic	8.33%	99.71%	50.00%	96.86%	1.088	28.354
Vaccination Status	History of Vaccination	4.17%	79.96%	0.46%	95.74%	0.834	0.208
	Not vaccinated or unknown history	95.83%	57.20%	4.73%	97.41%	13.718	2.239
Status of Dog at Time of Investigation	Found Alive	47.92%	2.36%	1.71%	58.18%	0.045	0.491
	Found Dead	52.08%	97.21%	39.68%	98.29%	2.029	18.654
Assessor’s Decision	Dog not showing signs of Rabies	2.08%	8.08%	0.08%	99.86%	1.020	14.496
	Dog likely to be rabid	79.17%	91.92%	33.04%	99.23%	4.764	102.446
	Dead/Not Assessed	18.75%	97.58%	21.43%	97.15%	1.196	6.572

**Table 4:** Risk matrix ^1^.

		Objective Risk Matrix								
Exposure Consideration	Probability of Rabies Based on Clinical Categorization of Bite (Babes)	Limited Surveillance Program Capacity							Advanced Surveillance Program Capacity	
		Dog Symptomatic	Dog Dead At Follow-up	Dog Bite Was Not Provoked	Stray Dog	Dog Bit Multiple People	Dog Not Vaccinated	Dog Healthy and Available for Quarantine	Dog Healthy 10 Days Post-Bite	Tested Negative
Bite to head/neck	45.00%	27.99%	17.87%	6.75%	6.26%	4.77%	2.12%	0.04%	0.00%	0.00%
Multiple severe bite wounds	27.50%	17.11%	10.92%	4.13%	3.82%	2.92%	1.29%	0.02%	0.00%	0.00%
Bites to young children	27.50%	17.11%	10.92%	4.13%	3.82%	2.92%	1.29%	0.02%	0.00%	0.00%
Bites to extremities	5.00%	3.11%	1.99%	0.75%	0.70%	0.53%	0.24%	0.00%	0.00%	0.00%
Minor bites (no break in skin)	1.00%	0.62%	0.40%	0.15%	0.14%	0.11%	0.05%	0.00%	0.00%	0.00%
Category II	1.00%	0.62%	0.40%	0.15%	0.14%	0.11%	0.05%	0.00%	0.00%	0.00%
*Medley* et al. *probability of rabies* ^2^		62.20%	39.70%	15.00%	13.90%	10.60%	4.70%	0.08%	0.00%	0.00%

^1^ Risk levels illustrated by Green (Low), Yellow/Orange (Moderate), and Red (High). ^2^ Probability of rabies as calculated from the data sets used in this study. “Dog Bite Was Not Provoked” was a best guess estimate at 15%.

## Supplementary Materials

The following are available online at www.mdpi.com/2414-6366/2/2/14/s1, Supplementary S1: HARSP Surveillance Form and Investigation Checklist. Supplementary S2: Proportion of Surviving Dogs during 14-Day Rabies Quarantine.

## References

[1] Hampson K., Coudeville L., Lembo T., Sambo M., Kieffer A., Attlan M., Barrat J., Blanton J.D., Briggs D.J., Cleaveland S. (2015). Estimating the global burden of endemic canine rabies. PLoS Negl. Trop. Dis..

[2] Tierkel E.S. (1975). Chapter 8: Canine rabies. The Natural History of Rabies.

[3] Wallace R.M., Reses H., Franka R., Dilius P., Fenelon N., Orciari L., Etheart M., Destine A., Crowdis K., Blanton J.D. (2015). Establishment of a canine rabies burden in Haiti through the implementation of a novel surveillance program. PLoS Negl. Trop. Dis..

[4] Rupprecht C.E., Briggs D., Brown C.M., Franka R., Katz S.L., Kerr H.D., Lett S.M., Levis R., Meltzer M.I, William Schaffner W. (2010). Use of a reduced (4-dose) vaccine schedule for postexposure prophylaxis to prevent human rabies: Recommendations of the Advisory Committee on Immunization Practices. MMWR Recomm. Rep..

[5] World Health Organization WHO Guide for Rabies Pre and Post Exposure Prophylaxis in Humans. Updates 2014. http://www.who.int/rabies/PEP_Prophylaxis_guideline_15_12_2014.pdf.

[6] World Health Organization (2014). WHO Expert Consultation on Rabies.

[7] Shantavasinkul P., Wilde H. (2011). Post-exposure prophylaxis for rabies in resource-limited/poor countries. Adv. Virus Res..

[8] Hampson K., Cleaveland S., Briggs D. (2011). Evaluation of cost-effective strategies for rabies post-exposure vaccination in low-income countries. PLoS Negl. Trop. Dis..

[9] Undurraga E.A., Wallace R.M., Blanton J.D., Cleaton J., Franka R. (2017). Elimination of dog-mediated human rabies deaths by 2030: Needs assessment and alternatives for progress based on dog vaccination. Front. Vet. Sci..

[10] Etheart M. 2017–currently in review with Lancet Global Health.

[11] Coetzer A., Kidane A.H., Bekele M., Hundera D.D., Pieracci E.G., Shiferaw M.L., Wallace R.M., Nel L.H. (2016). The SARE tool for rabies control: Current experience in Ethiopia. Antivir. Res..

[12] The Global Alliance for Rabies Control Website. rabiesalliance.org.

[13] Babes V. (1912). Traité de la Rage (Treatise on Rabies).

[14] Deeks J.J., Altman D.G. (2004). Statistics Notes: Diagnostic tests 4: Likelihood ratios. BMJ.

[15] Knust B., MacNeil A., Rollin P.E. (2011). Hantavirus pulmonary syndrome clinical findings: Evaluating a surveillance case definition. Vector Borne Zoonotic Dis..

[16] McGee S. (2002). Simplifying Likelihood Ratios. J. Gen. Intern. Med..

[17] Tepsumethanon V., Wilde H., Meslin F.X. (2005). Six criteria for rabies diagnosis in living dogs. J. Med. Assoc. Thail..

[18] Indrayan A. (2013). Basic Methods of Medical Research.

[19] Abela-Ridder B., Knopf L., Martin S., Taylor L., Torres G., De Balough K. (2016). 2016: The beginning of the end of rabies?. Lancet Glob. Health.

[20] Benotti P., Wood G.C., Winegar D.A., Petrick A.T., Still C.D., Argyropoulos G., Gerhard G.S. (2014). Risk factors associated with mortality after Roux-en-Y gastric bypass surgery. Ann. Surg..

[21] Sriaroon C., Sriaroon S., Svastijaya D., Pakamatz K., Wilde H. (2005). Retrospective: Animal attacks and rabies exposures in Thai children. Travel Med. Infect. Dis..

[22] Brown C.M., Slavinski S., Ettestad P., Sidwa T.J., Sorhage F.E. (2011). Compendium of animal rabies prevention and control: National Association of State Public Health Veterinarians, Inc.. J. Am. Vet. Med. Assoc..

[23] Tepsumethanon V., Wilde H., Sitprija V. (2008). Ten-day observation of live rabies suspected dogs. Dev. Biol. (Basel).

[24] Consales C.A., Bolzan V.L. (2007). Rabies review: Immunopathology, clinical aspects and treatment. J. Venom. Anim. Toxins Incl. Trop. Dis..

[25] Tipold A. (1995). Diagnosis of inflammatory and infectious diseases of the central nervous system in dogs: A retrospective study. J. Vet. Intern. Med..

[26] Taylor L.H., Knopf L. (2015). Partners for rabies prevention. surveillance of human rabies by national authorities-a global survey. Zoonoses Public Health.

