# Supplementary material for: Retrospective Cohort Study to Assess the Risk of Rabies in Biting Dogs, 2013–2015, Republic of Haiti
Source: Trop Med Infect Dis. 2017 Jun 12;2(2):14. doi: 10.3390/tropicalmed2020014 (PMC6082081; doi:10.3390/tropicalmed2020014)
Supplement: Supplementary file 1 [file tropicalmed-02-00014-s001.pdf]

# Supplementary Materials Materials: Retrospective Cohort Study to Assess the Risk of Rabies in Biting Dogs, 2013–2015, Republic of Haiti

**Alexandra M. Medley, Max Francois Millien, Jesse D. Blanton, Xiaoyue Ma, Pierre Augustin, Kelly Crowdis and Ryan M. Wallace**

**Supplementary S1.** HARSP Surveillance Form and Investigation Checklist.

**Date of Notification: _ _/ _ _ / _ _ _ _ Name ________________________ Animal ID: |__|__| |__|__| |__||__|**

Medical Contact Name: ____________________________

Phone Number: _________________

Patient Name: ____________________________________

Age ___________

Phone Number: _________________

1. Reported From: ☐ DELR → NSSS Number: |__|__|__|__|__|

☐ Health Department: ________________________

☐ Hospital: _________________________________

☐ Vet Agent: ______________________________

☐ Veterinarian

☐ Public

2. Reason for report: ☐ Human Exposure (bite or scratch) ☐ Sick animal ☐ Hit by car ☐ Other __________________

3. Type of animal: ☐ Dog ☐ Cat ☐ Goat ☐ Pig ☐ Mongoose ☐ Other ____________________________

4. Was this animal: ☐ Owned ☐ Stray ☐ Unknown

5. Location of animal exposure: Department__________________ Commune ______________________ Street_____________________

**NOTES**:

6. Date of Investigation: ______________ 7. Type of Investigation? ☐ Owner Phone Consultation ☐ In-Person Investigation

8. How many people were bitten by the animal? ______________ How many people were scratched by the animal? ______________

9. How many people already started rabies vaccine? ___________ How many people did you refer for medical treatment? __________

10. What other animals were bitten by this animal? How many? ☐ Dog _____ ☐ Cat _____ ☐ Goat _____ ☐ Other _____

11. Was the animal located? **Yes** ☐ Alive ☐ Escaped capture ☐ Dead, killed by owner ☐ Dead, killed by public
☐ Dead, killed by car ☐ Dead, natural causes ☐ Dead, unknown causes

**No** ☐ Not found ☐ Dead, killed by owner ☐ Dead, killed by public
☐ Dead, killed by car ☐ Dead, natural causes ☐ Dead, unknown causes

12. Where was animal located? Department/Commune__________________

13. What is the animal's age? ☐ Puppy ☐ Junior ☐ Adult ☐ Senior ☐ Unknown

14. What is the animal's sex? ☐ Male ☐ Female

15. Has the animal been vaccinated for rabies? ☐ Yes, year: _________ ☐ Not vaccinated ☐ Unknown

**NOTES**:

16. Signs of Disease: ☐ Aggression ☐ Biting ☐ Hypersalivation ☐ Paralyzed ☐ Lethargy ☐ Unknown

☐ Other (specify) ________________________________________________________________________________

17. Rabies Assessment: ☐ Healthy ☐ Sick, signs of rabies ☐ Sick, not rabies ☐ Dead

☐ Other (specify)________________________________________________________________________________

18. Assessment Decision: ☐ Quarantine ☐ Euthanize ☐ Dead ☐ Other ____________________________________________

19. Quarantine results: ☐ Healthy after 14 days ☐ Died, date _________ ☐ Euthanized ☐ Natural Causes ☐ Other

20. Was the animal submitted for testing? ☐ Yes, date: _________ ☐ No ☐ Animal was lost ☐ Decomposed / Burned ☐ Body discarded ☐ Other _______________

**NOT**



Day 5 follow up:

Day 10 follow up:

21. Date specimen received at lab: ______________ 22. Lab ID Number: ______________ 23. Date tested: ______________

24. Test Results: ☐ Positive ☐ Negative ☐ Inconclusive

☐ **Hospital notified, date_______________** ☐ **Health department notified, date _______________**

| **Investigation Checklist** | |
|---|---|
| **BEFORE DEPARTURE** | **NOTES** |
| Call owner, conduct verbal interview (Form 1a) | |
| Check inventory | |
| Verify equipment is in working condition | |
| Take necessary medications from the lock box | |
| Report your investigation destination to a colleague | |
| Plan route | |
| **UPON ARRIVAL** | **NOTES** |
| Locate the owner or bite victim | |
| Prevent crowds from gathering | |
| Ask for other animals to be kept away | |
| Locate the offending animal, if in the area | |
| Plan Escape Route | |
| Develop plan | |
| Does the animal need to be secured? | |
| Does the animal require sedation? | |
| Does the animal require euthanasia? | |
| Ready your equipment | |
| Ready your medications | |
| Ensure the area around the animal is safe and people and other animals are out of harm's way | |
| Secure the animal according to the plan developed | |
| Conduct investigation, complete the Form | |
| Conduct appropriate education with owners, victim and community | |
| Log Drugs Used in Log Book | |
| Ensure scene is clean, do not leave garbage or infectious materials | |
| **UPON RETURN** | **NOTES** |
| Process the animal for storage or testing | |
| Incinerate the body and disposable supplies that were used | |
| Organize supplies and equipment | |
| Return medications to lock box | |
| Review investigation form, complete as necessary | |
| Store forms in secured drawer | |

**Supplementary S2.** Proportion of Surviving Dogs during 14-Day Rabies Quarantine.

| Days Until Death | % Rabies Positive in Quarantine Still Alive | % Case Negative in Quarantine Still Alive | % of All Case Negatives Still Alive |
|---|---|---|---|
| 0 | 91.67 | 75 | 97.8 |
| 1 | 66.67 | 66.67 | 97.65 |
| 2 | 16.67 | 58.33 | 97.65 |
| 3 | 0 | 41.67 | 97.5 |
| 4 | 0 | 41.67 | 97.35 |
| 5 | 0 | 33.33 | 97.35 |
| 6 | 0 | 16.6 | 97.28 |
| 7 | 0 | 8.33 | 97.13 |
| 8 | 0 | 8.33 | 97.06 |
| 9 | 0 | 8.33 | 97.06 |
| 10 | 0 | 8.33 | 97.06 |
| 11 | 0 | 0 | 97.06 |
| 12 | 0 | 0 | 97.06 |
| 13 | 0 | 0 | 97.06 |
| 14 | 0 | 0 | 97.06 |
